# Exploration of 3D Few-Shot Learning Techniques for Classification of Knee Joint Injuries on MR Images

**DOI:** 10.3390/diagnostics15141808

**Published:** 2025-07-18

**Authors:** Vinh Hiep Dang, Minh Tri Nguyen, Ngoc Hoang Le, Thuan Phat Nguyen, Quoc-Viet Tran, Tan Ha Mai, Vu Pham Thao Vy, Truong Nguyen Khanh Hung, Ching-Yu Lee, Ching-Li Tseng, Nguyen Quoc Khanh Le, Phung-Anh Nguyen

**Affiliations:** 1 Department of Radiology, Pham Ngoc Thach University of Medicine, Ho Chi Minh City 740500, Vietnam; hiepdv@pnt.edu.vn; 2 Graduate Institute of Data Science, College of Management, Taipei Medical University, New Taipei City 23561, Taiwan; m946113015@tmu.edu.tw (M.T.N.); phatnguyenthuan1502@gmail.com (T.P.N.); 3 Graduate Institute of Biomedical Materials and Tissue Engineering, College of Biomedical Engineering, Taipei Medical University, New Taipei City 23561, Taiwan; 4 Research Center of Data Science on Healthcare Industry, College of Management, Taipei Medical University, New Taipei City 23561, Taiwan; quocviet09clc@gmail.com; 5 Department of Computer Science and Information Engineering, National Taiwan University, Taipei 10617, Taiwan; d10922024@ntu.edu.tw; 6 International Ph.D. Program in Medicine, College of Medicine, Taipei Medical University, Taipei 110301, Taiwan; d142111018@tmu.edu.tw; 7 Department of Radiology, Thai Nguyen National Hospital, Thai Nguyen City 24000, Vietnam; 8 Department of Orthopedic and Trauma, Cho Ray Hospital, Ho Chi Minh City 700000, Vietnam; drhung.bvcr@gmail.com; 9 Department of Orthopedics, Taipei Medical University Hospital, Taipei 110301, Taiwan; lee161022@tmu.edu.tw; 10 Department of Orthopaedics, School of Medicine, College of Medicine, Taipei Medical University, Taipei 110301, Taiwan; 11 International Ph.D. Program in Cell Therapy and Regenerative Medicine, College of Medicine, Taipei Medical University, Taipei 110301, Taiwan; 12 International Ph.D. Program in Biomedical Engineering, College of Biomedical Engineering, Taipei Medical University, New Taipei City 23561, Taiwan; 13 Research Center of Biomedical Device, College of Biomedical Engineering, Taipei Medical University, Taipei 110301, Taiwan; 14 In-Service Master Program in Artificial Intelligence in Medicine, College of Medicine, Taipei Medical University, Taipei 110301, Taiwan; khanhlee@tmu.edu.tw; 15 Translational Imaging Research Center, Taipei Medical University Hospital, Taipei 110301, Taiwan; 16 AIBioMed Research Group, Taipei Medical University, Taipei 110301, Taiwan; 17 Clinical Big Data Research Center, Taipei Medical University Hospital, Taipei 110301, Taiwan; 18 Clinical Data Center, Office of Data Science, Taipei Medical University, Taipei 110301, Taiwan

**Keywords:** knee injuries, MR images, few-shot learning, deep learning (DL), Generalized End-to-End (GE2E) loss

## Abstract

Accurate diagnosis of knee joint injuries from magnetic resonance (MR) images is critical for patient care. **Background/Objectives**: While deep learning has advanced 3D MR image analysis, its reliance on extensive labeled datasets is a major hurdle for diverse knee pathologies. Few-shot learning (FSL) addresses this by enabling models to classify new conditions from minimal annotated examples, often leveraging knowledge from related tasks. However, creating robust 3D FSL frameworks for varied knee injuries remains challenging. **Methods**: We introduce MedNet-FS, a 3D FSL framework that effectively classifies knee injuries by utilizing domain-specific pre-trained weights and generalized end-to-end (GE2E) loss for discriminative embeddings. **Results**: MedNet-FS, with knee-MRI-specific pre-training, significantly outperformed models using generic or other medical pre-trained weights and approached supervised learning performance on internal datasets with limited samples (e.g., achieving an area under the curve (AUC) of 0.76 for ACL tear classification with k = 40 support samples on the MRNet dataset). External validation on the KneeMRI dataset revealed challenges in classifying partially torn ACL (AUC up to 0.58) but demonstrated promising performance for distinguishing intact versus fully ruptured ACLs (AUC 0.62 with k = 40). **Conclusions**: These findings demonstrate that tailored FSL strategies can substantially reduce data dependency in developing specialized medical imaging tools. This approach fosters rapid AI tool development for knee injuries and offers a scalable solution for data scarcity in other medical imaging domains, potentially democratizing AI-assisted diagnostics, particularly for rare conditions or in resource-limited settings.

## 1. Introduction

Knee injuries are widespread across various age groups, including athletes, the elderly, and increasingly in children [1,2]. Nirav Maniar et al. reported the incidence of knee injuries in Australian patients [1], and anterior cruciate ligament (ACL) injuries were the most common with an annual growth rate of 7.3% to 10.4% in children. Other specific knee ligaments injuries such as the posterior cruciate ligament (PCL), medial collateral ligament (MCL), lateral collateral ligament (LCL), and meniscus tears were prevalent. Accurate quantitative assessment of knee joints in magnetic resonance (MR) images is crucial for studying and diagnosing injuries of the knee, a leading cause of disability in aged populations. However, accurate diagnosis of knee injuries still poses significant challenges due to complex anatomy [3,4] and indistinct lesion boundaries [5].

Deep learning (DL) techniques—particularly convolutional neural networks (CNNs)—have recently driven significant advancements in the classification and detection of knee injuries using both 2D and 3D MRI [6,7,8]. Bien et al. introduced the MRNet model, one of the early applications of deep learning for detecting ACL and meniscus tears, which utilized AlexNet to extract features from all three imaging planes (sagittal, coronal, and axial), thereby reducing the risk of missing injuries [9]. Liu et al. developed high-performing deep learning models for diagnosing ACL tears [10] and detecting cartilage lesions [11] with remarkable accuracy. Most studies primarily focused on anterior cruciate ligament (ACL) tears and meniscus injuries (cartilage), while other components of the knee remain underexplored. This limitation is partly due to the scarcity of annotated datasets and the substantial human effort required to label and to create high-quality datasets [7]. These challenges continue to constrain progress and hinder the development of more generalized and comprehensive models for knee injury analysis.

To the best of our knowledge, a few MRI datasets of the knee are publicly accessible. Specifically, the SKI10 dataset, one of the earliest public collections, was introduced at the Grand Challenge Workshop at MICCAI 2010 to facilitate the development of segmentation algorithms for cartilage and bone in MRI images, with a particular focus on knee osteoarthritis assessment [12]. In 2018, researchers from Stanford University released the MRNet dataset, aimed at supporting the training and evaluation of deep learning models for diagnosing knee injuries, including ACL tears, meniscus tears, and other abnormalities [9]. Additionally, the KneeMRI dataset, published in 2017 by the Clinical Hospital Centre Rijeka in Croatia, consisted of 917 MRI knee scans with annotations for anterior cruciate ligament (ACL) conditions, encompassing healthy states, partial tears, and complete tears [13]. These datasets have been instrumental in driving advancements in computer vision and machine learning research for knee injury assessment.

Prior studies have developed a range of deep learning techniques—including semi-supervised methods [7], transfer learning [8], generative adversarial networks (GANs) [14], few-shot learning [15], meta-learning [16], and imbalance learning [17]—to overcome data limitations. These approaches all share the advantage of reducing dependence on large annotated datasets for knee MRI analysis. Few-shot learning (FSL) algorithms have shown great promise for medical-imaging classification tasks that must recognize unseen classes from only a handful of examples [18,19,20]. In the few-shot learning (FSL) framework, the dataset is typically partitioned into a support set and a query set. The support set contains a limited number of labeled examples per class, which serve as reference points or prototypes, while the query set comprises unlabeled examples to be classified by measuring their similarity to these prototypes, often using embedding vectors. The parameter *k* refers to the number of labeled samples per class in the support set; hence, an FSL task with k samples per class is known as a *k*-shot task.

**Figure 1:**
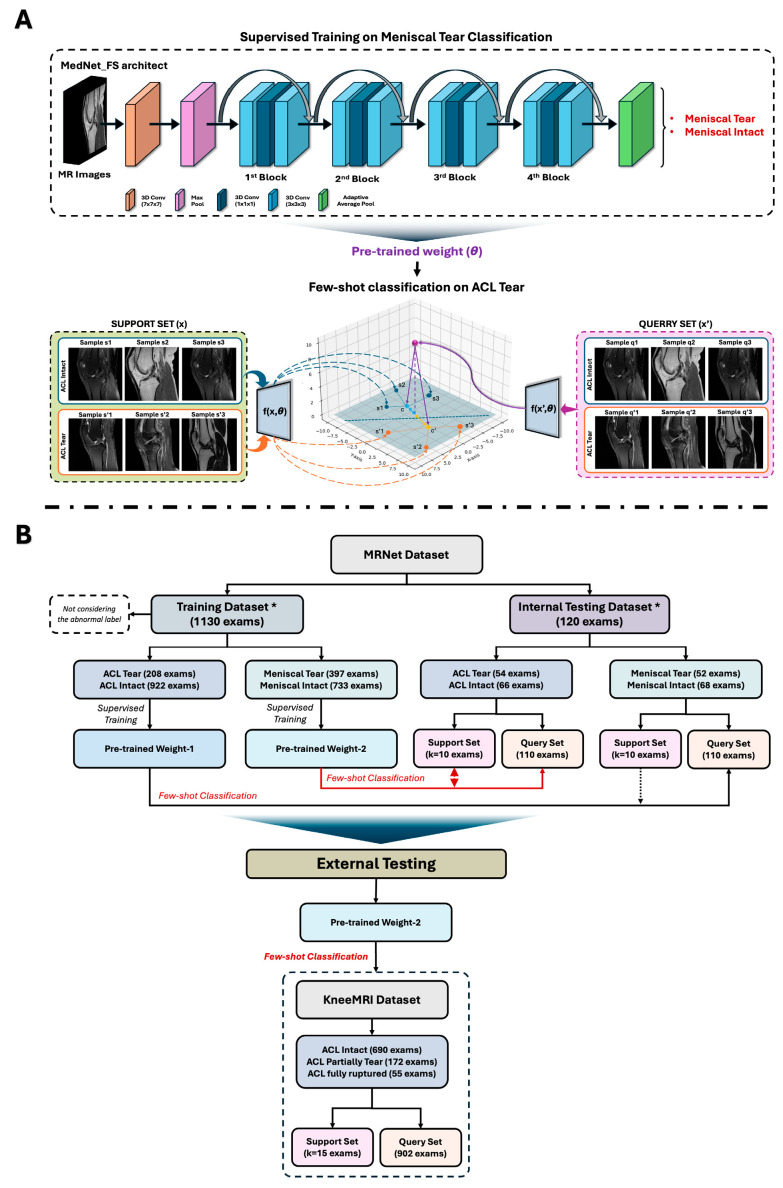
Overview of our study design and data usage. (**A**) Visualization of supervised training process with MedNet (ResNet101) framework on a specific knee injury with 3D MR images and utilization mechanism of few-shot learning algorithms by using its pre-trained weight to classify other knee injuries; (**B**) Our experimental setup flowchart to create distinct pre-trained weights in training, internal, and external testing process. * The division of training and testing datasets was based on the original data’s predefined classification.

In this study, we propose MedNet-FS, a framework that has utilized a few-shot algorithm and generalized end-to-end (GE2E) loss to perform binary classification of knee injuries on MR images (Figure 1A). We evaluated the diagnostic performance of the MedNet-FS model across multiple datasets, including public datasets, MRNet [9], and KneeMRI [13]. Moreover, a key aspect of few-shot learning algorithms has been leveraging suitable pre-trained weights to generate accurate embeddings from 3D MR images. We compared the performance of our custom pre-trained weights against various other pre-trained weights, including both general 3D weights and those specifically trained on 3D medical imaging.

## 2. Materials and Methods

### 2.1. Data Selection

In this study, we utilized two publicly available datasets, MRNet [9] and KneeMRI [13]. The MRNet dataset [9] was used for both training and internal testing of the MedNet-FS model. This dataset consists of 1370 MRI exams of the knee, aimed at detecting lesions such as ACL tears (AT), meniscus tears (MT), and other abnormalities. Each exam includes three series of images (sagittal, coronal, and axial planes), with each image typically sized at 256 × 256 pixels.

To validate the performance of MedNet-FS, we employed the KneeMRI dataset [13] that was collected at the Clinical Hospital Centre Rijeka in Croatia, spanning the period from 2006 to 2014. This dataset comprised 917 volumes, with diagnoses regarding ACL conditions categorized into three classes: (1) healthy, (2) partially injured, and (3) completely ruptured, determined through a double-blind labeling process. All MRI scans were obtained before surgery, with arthroscopic knee surgery results serving as the ground truth. A summary of the three datasets is presented in Table 1.

### 2.2. Data Usage

In this study, we employed a training process on the pre-split training and test sets of the MRNet dataset (Figure 1B) [9]. The procedure was sequential and tailored to specific few-shot learning targets (Figure 1A). We prepared two different sets of pre-trained weights:

- Pre-trained Weight 1: obtained from supervised training to distinguish ACL tear from intact ACL.
- Pre-trained Weight 2: obtained from supervised training to distinguish meniscal injuries from non-meniscal injuries.

These training processes were conducted independently for each target, ensuring distinct and focused model development. The resulting pre-trained weights were then used for few-shot classification of knee injuries in MR images. In this study, we specifically conducted a binary classification task (2-way), implemented under a *k*-shot configuration. Here, kkk denotes the number of labeled examples per class in the support set, explicitly set to k = 2, 20, and 40. Furthermore, the training and testing sets were completely disjointed, ensuring patient-level separation to avoid data leakage and guarantee unbiased model evaluation. The predicted labels were compared against the ground truth to evaluate model performance. We applied our MedNet-FS model, initialized with the above pre-trained weights, to perform few-shot classification on internal and external test sets (Figure 1B).

Notably, although the KneeMRI dataset focuses on ACL tear classification, we opted to use the “Pre-trained Weight 2” (trained on meniscus tear vs. non-meniscus tear). This approach allows us to test the transferability of features learned from a *related but distinct* knee injury classification task, thereby avoiding potential overfitting or bias that might arise if the model had already been trained directly on ACL classification. By using meniscus-based pre-trained weights, we aim to demonstrate the model’s ability to adapt to new tasks under a few-shot learning framework. In addition to the MRNet pre-trained weights, we also utilized two other types of pre-trained models to evaluate the robustness of our few-shot framework. The first is a general pre-trained weight, primarily trained on video recognition datasets [21], which captures spatiotemporal features common in volumetric data. The second is a medical image pre-trained weight derived from non-knee MRI datasets [22], allowing us to assess transferability from broader medical imaging contexts. These variations help us explore how different pre-training sources influence downstream few-shot performance on knee MRI classification tasks.

### 2.3. MedNet-FS Model Construction

In this study, we proposed the MedNet-FS model, a few-shot learning approach that integrated a modified MedNet (based on ResNet101) [23] with the generalized end-to-end (GE2E) loss function. To adapt MedNet for few-shot tasks, we replaced its final linear layer with an adaptive pooling layer to generate robust embedding vectors, thereby removing the need for a dedicated classifier. Specifically, the few-shot learning framework was built on a prototype network that incorporated a pre-trained model. First, embeddings for the support set were generated using the pre-trained model. For each label, we computed a prototype by taking the mean of its corresponding embedding vectors. A query sample was then classified by identifying the label whose prototype had the smallest Euclidean distance to the sample’s embedding.

MedNet-FS was trained using the GE2E loss function [24]. This loss was designed to pull embeddings of samples from the same label closer together while pushing those from different labels further apart. Formally, let each embedding vector be denoted as f_ij_, which means the feature extracted from the j_th_ sample of i_th_ label. S_ji_, k is the cosine similarity between embedding vector f_ij_ to centroids C_k_ of the kth label. The loss function on each embedding vector can be defined as:

(1) $$L_{e_{ij}}=-S_{ji,i}+log\sum_{k=1}^{n}e^{S_{ji,k}},$$

This formulation not only mitigated the influence of a final linear classifier layer but also enabled the model to learn effective clustering of embedding vectors, a key requirement for few-shot classification.

Additionally, we evaluated the performance of our approach by comparing it with renowned multi-3D-CNN frameworks, including DenseNet [25], VGG [26], and AlexNet [27], which served as backbones for generating the pre-trained weights of the prototype network. The experiments were conducted on an RTX A4000 GPU (12 Gb), batch size = 8, epochs, optimizers, learning rate, and number of parameters are detailed in Table S2.

### 2.4. Performance Metrics

Our model was primarily evaluated using four metrics: sensitivity (SEN), specificity (SPE), accuracy (ACC), and the area under the receiver operating characteristic curve (ROC-AUC). These metrics were applied to the public test set, MRNet validation dataset.

### 2.5. Statistical Analysis

Statistical analysis was performed using GraphPad Prism version 10.3.1 for Mac (GraphPad Software, San Diego, CA, USA) [28]; *p* < 0.05 (*), 0.01 (**), 0.001 (***), 0.0001 (****) indicated a significant difference. Multiple *t*-tests with 95% confidence intervals (CIs) were used to evaluate differences in model performance.

## 3. Results

### 3.1. Patient Characteristics

For training and internal testing, this study utilized the MRNet dataset, comprising 1250 knee MRI scans from 1199 patients. According to Table 1, the patients in this dataset had an average age of 38.11 (±16.90) years; BMI information was not available. In line with our study design, the MRNet dataset was stratified into cases for ACL tear versus non-ACL tear classification and meniscal tear versus non-meniscal tear classification.

External validation was performed using the KneeMRI dataset, which included 917 MRI scans. This dataset was primarily employed for classifying ACL conditions—encompassing partially torn, fully ruptured, and intact ACLs—against normal findings. Both the MRNet and KneeMRI datasets present demographic and imaging variability, including the use of different MRI scanners (GE scanners at 1.5 T and 3.0 T for MRNet and a Siemens Avanto 1.5 T scanner for KneeMRI). This heterogeneity was leveraged to assess the generalizability of our proposed MedNet-FS approach. Further details, including specific class distributions and scanner types for each dataset, are summarized in Table 1. Detailed MRI scanning parameters for these datasets are provided in Table S1.

### 3.2. Investigation of Various Pre-Trained Weights and CNN Frameworks in Few-Shot Classification of Knee Injuries

**Figure 2:**
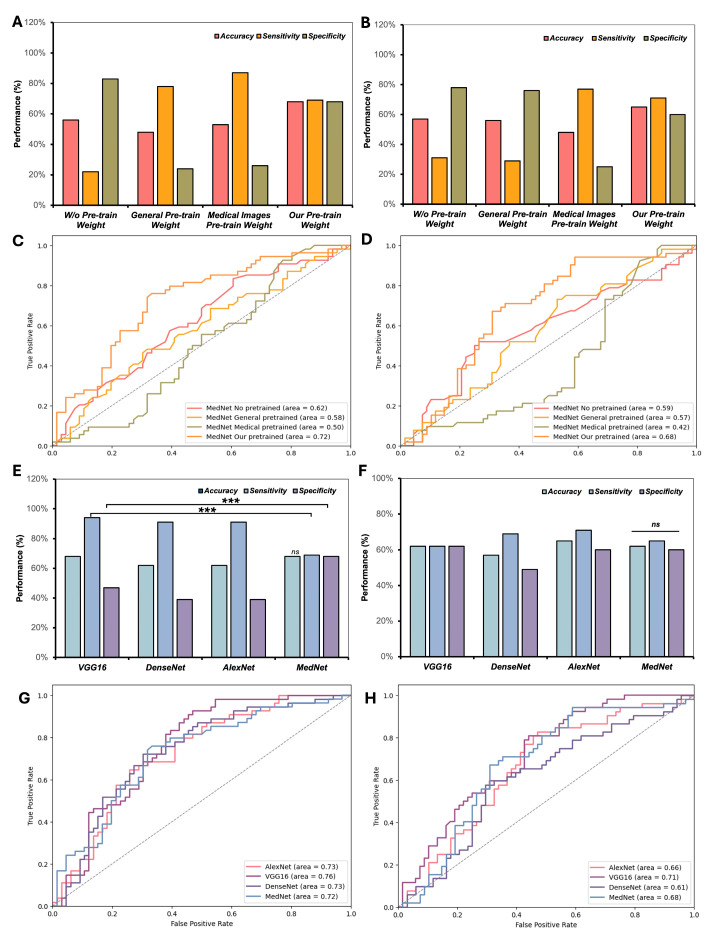
Comparative performance of different pre-trained weight strategies and CNN frameworks for few-shot classification of ACL and meniscal tears on the MRNet dataset. (**A**,**B**) illustrate performance metrics (accuracy, sensitivity, specificity) for ACL and meniscal tear classification, respectively, using various pre-trained weights with the MedNet framework. Correspondingly, (**C**,**D**) present the ROC-AUC curves for these pre-trained weight comparisons. Subsequently, (**E**,**F**) display performance metrics for ACL and meniscal tear classification using different CNN frameworks (e.g., MedNet, VGG16, DenseNet, AlexNet) initialized with custom pre-trained weights, while (**G**,**H**) illustrate their respective ROC-AUC curves. (*** *p* < 0.001, significant different; ns, no significant).

In this study, we explored the influence of different pre-trained weight configurations on few-shot classification of knee injuries (Figure 2A–D). Notably, our custom pre-trained weights, derived from knee MR images labeled for a single condition, demonstrated substantial advantages when predicting other injuries on the MRNet dataset [9]. For instance, pre-trained weights derived from ACL tear images effectively facilitated the few-shot classification of meniscus tears and vice versa. Figure 2A shows that with our Pre-trained Weight 2, the MedNet model achieved an accuracy (ACC) of 0.68 and an AUC of 0.72 (Figure 2A,C) for ACL tear classification. For meniscus tear classification, the model achieved an ACC of 0.65 and an AUC of 0.68 (Figure 2B,D). In contrast, medical pre-trained weights yielded significantly lower performance, with MedNet achieving an ACC of 0.53 and an AUC of 0.50 for ACL tear classification (Figure 2A,C) and an ACC of 0.48 with an AUC of 0.42 for meniscus tear classification (Figure 2B,D). These results underscore the importance of pre-training on knee-specific data to optimize the effectiveness of few-shot learning for knee injury classification.

Additionally, the performance of different CNN frameworks for few-shot classification was also evaluated (Figure 2E–H). MedNet consistently outperformed other frameworks, including VGG16 (SEN: 0.94, SPE: 0.47), DenseNet (SEN: 0.74, SPE: 0.51), and AlexNet (SEN: 0.91, SPE: 0.39), providing the most balanced and stable results for ACL tear classification with an SEN of 0.69 and SPE of 0.68 (Figure 2E). For meniscus tear classification, MedNet achieved the best balance with an SEN of 0.71 and an SPE of 0.60, while VGG16 (SEN: 0.62, SPE: 0.62) and AlexNet (SEN: 0.65, SPE: 0.60) exhibited less balanced performance (Figure 2F). DenseNet offered slightly better balance than VGG16 and AlexNet but still underperformed compared to MedNet. Overall, MedNet demonstrated superior performance and stability, confirming its effectiveness as the most reliable framework for few-shot classification of ACL and meniscus tears in the MRNet dataset.

In addition, we also compared different CNN frameworks for their effectiveness in few-shot classification (Figure 2B). MedNet consistently outperformed other frameworks, including VGG16, DenseNet, and AlexNet, providing the most stable and balanced performance across both ACL tear and meniscus tear classification tasks. For ACL tear classification, MedNet achieved an SEN of 0.69 and an SPE of 0.68, outperforming VGG16 (SEN: 0.94, SPE: 0.47) and AlexNet (SEN: 0.91, SPE: 0.39) (Figure 2E). Similarly, for meniscus tear classification, MedNet delivered the best balance with an SEN of 0.71 and an SPE of 0.60, while VGG16 (SEN: 0.62, SPE: 0.62) and AlexNet (SEN: 0.65, SPE: 0.60) showed greater imbalance (Figure 2F). DenseNet provided slightly better balance than VGG16 and AlexNet but still underperformed compared to MedNet. Overall, MedNet demonstrated superior performance and stability, confirming its effectiveness as the most reliable framework for few-shot classification of ACL tears and meniscus tears on the MRNet dataset.

### 3.3. Performance of the MedNet-FS Model with GE2E Loss

**Figure 3:**
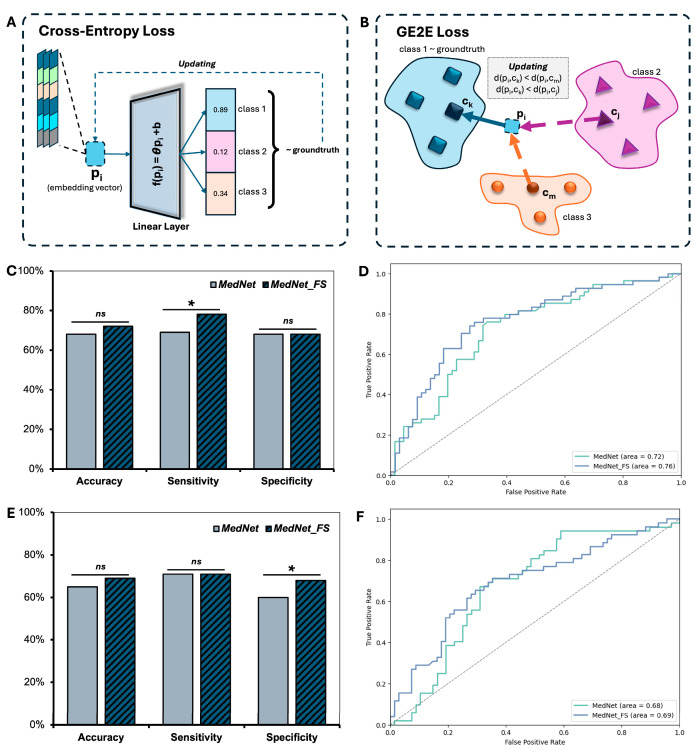
Impact of GE2E Loss on the few-shot classification performance of MedNet-FS. (**A**) Schematic illustration of the traditional Cross-Entropy Loss mechanism; (**B**) Schematic illustration of the GE2E Loss mechanism, designed to optimize the embedding space by enhancing intra-class compactness and inter-class separability; (**C**) Comparison of performance metrics (accuracy, sensitivity, specificity) between MedNet (baseline) and MedNet-FS (utilizing GE2E Loss) for ACL tear versus non-ACL tear classification; (**D**) Corresponding ROC-AUC curves for ACL tear classification, comparing MedNet and MedNet-FS; (**E**) Comparison of performance metrics between MedNet and MedNet-FS for meniscus tear versus non-meniscus tear classification; (**F**) Corresponding ROC-AUC curves for meniscus tear classification, comparing MedNet and MedNet-FS. (* *p* < 0.05, significant different; ns, no significant).

GE2E loss served as a key component in advancing the performance of few-shot classification models for knee injuries in our work. Figure 3 illustrates the mechanisms of cross-entropy loss and GE2E loss, highlighting their roles in optimizing feature embeddings. The traditional cross-entropy loss (Figure 3A) updates embedding vectors by minimizing prediction error relative to the ground truth label, yet it lacks the ability to explicitly manage intra-class compactness and inter-class separability. In contrast, GE2E loss (Figure 3B) enhances the embedding space by minimizing intra-class distances while maximizing inter-class distances, resulting in more discriminative and robust feature representations.

The impact of GE2E loss is demonstrated in the comparison between MedNet and MedNet-FS. For ACL tear versus non-ACL tear classification (Figure 3C,D), MedNet-FS achieves significantly higher sensitivity (*p* < 0.05) and achieved a higher area under the curve (AUC = 0.76) compared to MedNet (AUC = 0.72). Similarly, for meniscus tear versus non-meniscus tear classification (Figure 3E,F), MedNet-FS significantly improves specificity (*p* < 0.05) while also achieving a slightly higher AUC (0.69 vs. 0.68). These findings underscore the ability of GE2E loss to enhance the model’s capacity to distinguish between classes while maintaining a balanced trade-off between sensitivity and specificity. Overall, the integration of GE2E loss represents a critical advancement in addressing the challenges of few-shot classification, contributing to the development of a more reliable and generalizable model for knee injury classification.

### 3.4. Effectiveness of Few-Shot Learning Technique Compared to Supervised Learning on Small Samples

The performance of our MedNet-FS model was also compared with a supervised training model using the same framework and input data equivalent to k = 40 (20 injured cases and 20 normal cases). The results highlight the potential of few-shot learning to approach or exceed supervised learning performance as the number of support samples increases (Table 2). For ACL tear classification, the supervised MedNet-FS achieved an accuracy (ACC) of 0.67 and an area under the curve (AUC) of 0.75. The few-shot model, with fewer support samples, initially underperformed, achieving 0.43 ACC and 0.31 AUC with k = 2. However, its performance improved significantly as k increased, reaching 0.68 ACC and 0.70 AUC at k = 20 and achieving 0.72 ACC and 0.76 AUC at k = 40, surpassing the supervised model. A similar pattern was observed for meniscus tear classification. The supervised model achieved an ACC of 0.67 and an AUC of 0.64, while the few-shot model achieved 0.39 ACC and 0.25 AUC at k = 2, which improved to 0.73 ACC and 0.76 AUC at k = 20 and performed comparably to the supervised model with 0.69 ACC and 0.70 AUC at k = 40. These findings underscore the effectiveness of few-shot learning, which, with a small number of support samples, approaches or exceeds the performance of fully supervised models trained on equivalent data. Notably, as k increases, the few-shot model achieves higher sensitivity and specificity, highlighting its strong potential for generalization in low-data scenarios.

### 3.5. Performances of MedNet-FS Model on External Testing Datasets

**Figure 4:**
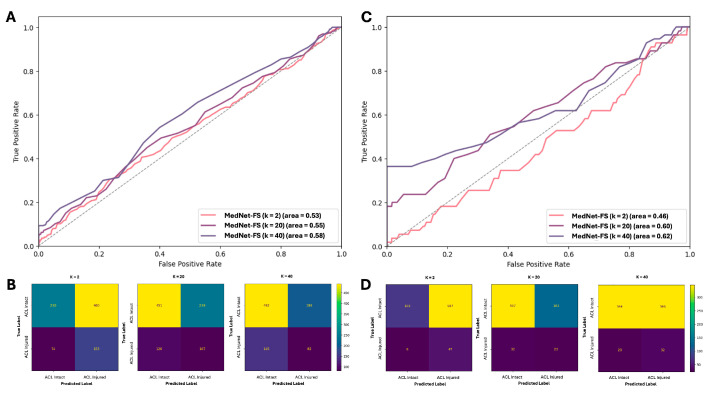
External validation of MedNet-FS for classifying ACL tear versus intact ACL on the KneeMRI dataset. (**A**) ROC-AUC curve and (**B**) confusion matrix illustrating the performance of the MedNet-FS model when validated with partially torn ACL cases included. (**C**) ROC-AUCcurve and (**D**) confusion matrix illustrating the performance of the MedNet-FS model when validated without partially torn ACL cases included.

This section presents the external validation results of our few-shot learning model, MedNet-FS (pre-trained on meniscus injury classification), for the binary classification of ACL tears versus intact ACLs using the KneeMRI dataset. The model’s performance was assessed with varying numbers of support cases (k = 2, 20, 40). Initial evaluations encompassed all presentations of ACL tears, including partially torn cases. As detailed in Table 3 (under “Including partially torn cases”), the model’s performance with k = 2 support samples resulted in an accuracy of 0.42, a sensitivity of 0.67, and a specificity of 0.33. With an increase in support samples to k = 20, accuracy rose to 0.63 (sensitivity 0.36, specificity 0.71), and at k = 40, accuracy was 0.61 (sensitivity 0.47, specificity 0.65). The area under the curve (AUC) showed a progression from 0.53 (k = 2) to 0.58 (k = 40). These initial outcomes were deemed suboptimal, a challenge attributed to the inherent difficulty and high prevalence of partially torn ACLs, especially given the reliance on vector embeddings from the few-shot model for classification. The performance under this scenario, including the discriminative ability reflected by the ROC-AUC curve and the specific error patterns from the confusion matrix, is visually summarized in Figure 4A and Figure 4B, respectively.

Given these challenges, a subsequent experiment was conducted by focusing the classification task on more distinct categories: intact ACLs versus fully ruptured ACLs, thereby excluding the ambiguous partially torn cases (Table 3, “Excluding partially torn cases”). This refined approach led to more encouraging results. While at k = 2, sensitivity was high (0.85) but specificity was very low (0.15), a significant improvement was observed at k = 20, with accuracy reaching 0.71 (sensitivity 0.42, specificity 0.73) and an AUC of 0.60. Most notably, with k = 40 support samples, this focused approach yielded an AUC of 0.62 (accuracy 0.50, sensitivity 0.58, specificity 0.50). This AUC value indicates a more promising discriminative capability for the MedNet-FS model when applied to clearly differentiated ACL states. Figure 4C (ROC-AUC curve) and 4D (confusion matrix) provide a visual representation of this improved performance, illustrating the enhanced class separation and classification accuracy achieved under this condition for a representative k setting.

## 4. Discussion

This study introduced MedNet-FS, a 3D few-shot learning (FSL) framework, to address the pressing clinical need for accurate and data-efficient classification of knee joint injuries from MR images. The potential key of clinical utility was the demonstration that the domain-specific pre-trained weights (from knee MRI images) were crucial for effective feature extraction, significantly outperforming generic or non-knee-specific medical pre-training. Furthermore, the integration of GE2E loss enhanced the discriminative power of embeddings, a vital factor for diagnostic confidence in clinical decision support. Notably, MedNet-FS achieved performance comparable to or exceeding supervised methods in low-data scenarios (support samples, k = 40), underscoring few-shot learning’s capacity to make advanced AI tools more accessible for various clinical conditions, even those with limited available data. 

The external validation of MedNet-FS for ACL tear classification on the KneeMRI dataset highlighted both its current capabilities and areas for refinement in a clinical context. Initial evaluations including partially torn ACLs yielded suboptimal AUCs (up to 0.58 at k = 40, Figure 4A,B), reflecting the inherent clinical difficulty in diagnosing these ambiguous injuries, a challenge amplified for few-shot learning (FSL) models reliant on global embeddings from limited slices depicting the ACL [29,30]. However, by focusing the task on more clinically distinct categories, intact versus fully ruptured ACLs (excluding partial tears), MedNet-FS demonstrated significantly more promising results, achieving an AUC of 0.62 at k = 40 (Figure 4C,D). This suggested that, while the model, in its current iteration, might require further development for subtle partial tears, it held immediate potential as a preliminary screening tool or a diagnostic aid for clear-cut ACL injuries [7], potentially streamlining clinical workflows [30].

Comparing these findings to the broader landscape, FSL in medical imaging has garnered increasing attention, with numerous studies exploring diverse applications across various pathologies and imaging modalities [20,31,32]. These works have demonstrated the potential of FSL for disease classification from limited medical datasets. However, it is crucial to emphasize that there is still a relative scarcity of research conducting few-shot classification directly on 3D medical image data, particularly magnetic resonance imaging (MRI) or computed tomography (CT) scans [15,33]. This scarcity is partly due to the inherent challenges associated with high data dimensionality and the complexity of anatomical structures in volumetric data. In this context, the AUC of 0.62 achieved by MedNet-FS in this study for distinguishing between intact and fully ruptured ACLs is considered comparable and competitive with results reported in contemporary few-shot classification studies in medical imaging, especially when considering the complexity of 3D knee MRI data.

MedNet-FS’s architecture offers several avenues for enhancing medical diagnostics. It could serve as a decision support system for radiologists and orthopedic specialists, particularly in high-volume or resource-limited settings where expert subspecialist availability might be constrained [34,35]. It may also act as a valuable research accelerator, enabling the rapid development of classifiers for less common knee pathologies with minimal data investment. Despite its strengths, such as being a pioneering 3D FSL approach for diverse knee injuries and comprehensively evaluating key FSL components, clinical adoption requires addressing limitations. These primarily include improving performance on ambiguous intermediate pathological states like partial tears and ensuring robust validation across diverse patient demographics and imaging hardware commonly found in clinical practice. Furthermore, seamless integration into existing hospital information systems and PACS is crucial for real-world utility. Future efforts should prioritize enhancing the clinical applicability of MedNet-FS. This involves research into advanced techniques to improve diagnostic accuracy for subtle injuries, potentially by incorporating segmentation to focus on relevant anatomy or by integrating multi-modal data [36,37]. The model’s limited performance on ambiguous partial ACL tears presents a key barrier to clinical translation, largely due to the subtle imaging features and insufficient representation in the current dataset. Addressing this issue will require expanding both the size and diversity of training data to ensure broader generalizability. Future research should also explore multi-modal data fusion (e.g., integrating clinical notes or radiology reports), semi-supervised learning to leverage large unlabeled datasets, and the incorporation of explainable AI (XAI) to enhance transparency and trust in model predictions. Developing clinician-friendly interfaces with XAI components will facilitate effective human–AI collaboration. Ultimately, prospective clinical studies are essential to assess the real-world impact of MedNet-FS on diagnostic accuracy and workflow efficiency. Continued collaboration between AI researchers and clinicians will be critical in refining few-shot learning models to better meet clinical demands and support improved patient outcomes.

## 5. Conclusions

This study successfully developed and validated MedNet-FS, a 3D few-shot learning framework for classifying knee joint injuries from MR images, effectively addressing the critical challenge of data scarcity in medical AI. Key findings underscore the necessity of domain-specific (knee MRI) pre-trained weights and the benefits of GE2E loss for creating highly discriminative embeddings, enabling MedNet-FS to achieve performance comparable to supervised methods even with minimal task-specific data. External validation on the KneeMRI dataset for ACL tear classification, while highlighting difficulties with ambiguous partially torn cases (AUC up to 0.58, k = 40), demonstrated promising results (AUC of 0.62, k = 40) when differentiating between distinct categories like intact versus fully ruptured ACLs. Overall, MedNet-FS presented a significant advancement by reducing dependency on extensive annotated datasets, paving the way for more accessible, adaptable, and rapidly deployable AI-driven diagnostic tools for a range of knee injuries and potentially other medical imaging applications, ultimately contributing to enhanced patient care.

## Figures and Tables

**Table 1:** Summary statistics of training, internal testing, and external testing datasets.

Datasets Name	MRNet [9]	KneeMRI [13]
Usage	Training/Internal testing	External testing
Total of exams	1250 exams	917 exams
Number of patients	1199 patients	N/A
Number of women (%)	530 (44.20)	N/A
Age, mean (SD)	38.11 (16.90)	N/A
BMI (kg/m^2^), mean (SD)	N/A	N/A
Source	Stanford University Medical Center, Stanford, CA, USA	Clinical Hospital Centre Rijeka, Rijeka, Croatia
Scanner	GE scanner (GE healthcare)	Siemens Avanto scanner (Siemens Healthineers)
Magnetic field	1.5 T and 3.0 T	1.5 T
Number of classes	3 classes	3 classes
Class distribution: case number (%)	- ACL tear: 262 (20.96%)	- Intact ACL: 690 (75.25%)
	- Meniscal tear: 449 (35.92%)	- ACL partial tear: 172 (18.76%)
	- Abnormal: 1008 (80.64%)	- ACL completely ruptured: 55 (5.99%)

N/A: Not Applicable.

**Table 2:** Comparing supervised learning and few-shot learning (k = 2, 20, 40) for classifying ACL and Meniscus Injuries.

Model	Classification Target	ACC	SEN	SPE	AUC
Supervise training ^1^	ACL Tear	0.67	0.48	0.79	0.75
	Meniscus Tear	0.67	0.32	0.93	0.64
MedNet-FS (k = 2)	ACL Tear	0.43	0.22	0.57	0.31
	Meniscus Tear	0.39	0.13	0.61	0.25
MedNet-FS (k = 20)	ACL Tear	0.68	0.65	0.72	0.70
	Meniscus Tear	0.73	0.72	0.74	0.76
MedNet-FS (k = 40)	ACL Tear	0.72	0.73	0.72	0.76
	Meniscus Tear	0.69	0.67	0.72	0.70

^1^ The supervised learning process was trained with the same support sample (k = 40) of few-shot learning process.

**Table 3:** Performance of our MedNet-FS dataset in binary classifying of ACL injury on KneeMRI.

	Initial Input Sample (k)	ACC	SEN	SPE	AUC
Including partially torn cases	k = 2	0.42	0.67	0.33	0.53
	k = 20	0.63	0.36	0.71	0.55
	k = 40	0.61	0.47	0.65	0.58
Excluding partially torn cases	k = 2	0.20	0.85	0.15	0.46
	k = 20	0.71	0.42	0.73	0.6
	k = 40	0.50	0.58	0.50	0.62

## Data Availability

Publicly available datasets were analyzed in this study. This data can be found here: MRNet [9] and KneeMRI datasets [13].

## Supplementary Materials

The following supporting information can be downloaded at: https://www.mdpi.com/article/10.3390/diagnostics15141808/s1, Table S1. Parameters of MRI Scanners in MRNet and KneeMRI Datasets; Table S2. Hyperparameters of MedNet-FS and other benchmarking models.

## Abbreviations

The following abbreviations are used in this manuscript:

ACL	Anterior cruciate ligament
PCL	Posterior cruciate ligament
MCL	Medial collateral ligament
LCL	Lateral collateral ligament
MR	Magnetic resonance
MRI	Magnetic resonance image
CNN	Convolutional neural network
DL	Deep learning
GE2E	Generalized end-to-end
ACC	Accuracy
AUC	Area under the curve
SEN	Sensitivity
SPE	Specificity
ROC-AUC	Area under the receiver operating characteristic curve
XAI	Explainable AI
FSL	Few-shot learning
SAG	Sagittal
