# Supplementary material for: Exploration of 3D Few-Shot Learning Techniques for Classification of Knee Joint Injuries on MR Images
Source: Diagnostics (Basel). 2025 Jul 18;15(14):1808. doi: 10.3390/diagnostics15141808 (PMC12293373; doi:10.3390/diagnostics15141808)
Supplement: Supplementary file 1 [file diagnostics-15-01808-s001.zip › diagnostics-3676046-supplementary.pdf]

Supplementary Data

Table S1. Parameters of MRI Scanners in MRNet and KneeMRI Datasets.

Dataset	Magnet	Protocol	Slice thick (mm)	Slice gap (mm)	Acquisition matrix size	Reconstructed matrix size	Bit depth
MRNet	3T	Sag PD	2.5	0	384 x 224 x 42	512 x 512 x 42	16
	3T	Sag T2 FS	2.5	0	384 x 192 x 42	512 x 512 x 42	16
	1.5T	Sag PD	3	1	512 x 192 x 24	512 x 512 x 24	16
	1.5T	Sag T2 FS	3.5	0.5	448 x 192 x 24	512 x 512 x 24	16
KneeMRI	1.5T	Sag PD	3	3.6	320 x 320 x 32	320 x 320 x32	12

Table S2. Hyperparameters of MedNet-FS and other benchmarking models.

Model	No. of. Params	Optimizers	Epoch	Learning rate	Loss function
MedNet_FS	85,201,729	Adam	100	0.0001	GE2E
MedNet	85,201,729	Adam	100	0.0001	Cross entropy
VGG16	188,072,514	Adam	100	0.0001	Cross entropy
AlexNet	175,148,866	Adam	100	0.0001	Cross entropy
DenseNet	12,267,624	Adam	100	0.0001	Cross entropy
